# Rate dependence of cell-to-cell variations of lithium-ion cells

**DOI:** 10.1038/srep35051

**Published:** 2016-10-11

**Authors:** Fuqiang An, Lufan Chen, Jun Huang, Jianbo Zhang, Ping Li

**Affiliations:** 1 Institute for Advanced Materials and Technology, University of Science and Technology Beijing, Beijing 100083, China; 2 Boston-Power Battery, Inc. Westborough, USA; 3 State Key Laboratory of Automotive Safety and Energy, Department of Automotive Engineering, Tsinghua University, Beijing 100084, China; 4 Beijing Co-innovation Center for Electric Vehicles, Beijing Institute of Technology, Beijing 100081, China

## Abstract

Lithium-ion cells are commonly used in a multicell configuration in power devices and electric vehicles, making the cell-to-cell variation (CtCV) a key factor to consider in system design and management. Previous studies on CtCV have two major limitations: the number of cells is usually less than one hundred, and the cells are usually commercial cells already subjected to cell-screenings. In this article, we first make a statistical analysis on the CtCV of 5473 fresh cells from an automotive battery manufacturer before the cell-screening process. Secondly, 198 cells are randomly selected from these 5473 cells and the rate dependence of the CtCV is examined, focusing on the correlations of capacity versus weight and capacity versus resistance, corresponding to thermodynamic and kinetic factors, respectively. The rate dependence of these two correlations is explained from a phenomenological model. Finally, eight cells from the 198 cells are further characterized with electrochemical impedance spectroscopy method to elucidate the kinetic origins of the CtCV.

Improving rate capability of lithium-ion cells (LICs) is a long-pursued task. In doing so, structural design and optimization play an important role that complements material innovations^1^,^2^. There have been considerable efforts in exquisitely tailoring the electrode structural parameters, viz. the thickness, the porosity and its gradient, to enhance the rate capability without sacrificing the energy density^1^,^2^. Structure-dependence of the rate capability has been revealed^1^,^2^. Particularly, the ion transport in the electrolyte phase is found to be the limiting factor in the rate capability of thick LIC electrodes^3^,^4^. Most of these efforts are on the scale of an electrode, or a single cell.

Scaling up from one cell to a module or pack brings into play the cell to cell variations (CtCVs) as a key concern^5^,^6^,^7^,^8^. However, little is known about the parameter space that governs the CtCVs. Two important questions are: how large are the contributions of thermodynamic and kinetic factors to the total CtCVs, and what role does the rate play in the competition between these two aspects? Understandings towards these two issues should lead to strategies for enhancing the CtCVs performance. Dubarry, Vuillaume and Liaw^9^ reported first attempts to separate the origins of CtCVs into three aspects: the amount of active material, polarization resistance, and localized kinetic factors, through statistically analyzing 100 cells in terms of their capacities, resistance, and incremental capacity curves. However, the rate dependence of CtCVs is not explored. This is the gap that this study aims to fill.

In what follows, we first give a statistical analysis of 5473 cells with a nominal capacity of 5.3 Ah. Then, 198 cells are selected and subjected to rate capability measurements, with special attention paid to the correlation of capacity versus weight and that of capacity versus resistance and their rate dependence behaviors. Finally, eight of them are further characterized with the electrochemical impedance spectroscopy method, so as to decipher the kinetic factors in greater details.

## Results

### Statistics of 5473 cells

**Figure 1:**
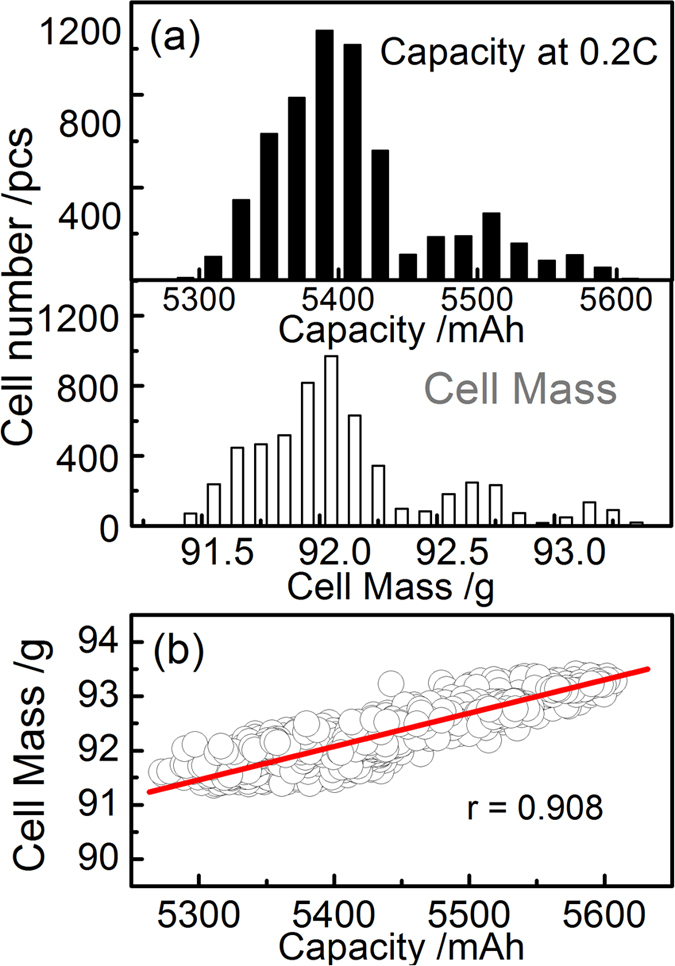
(**a**) Distributions of cell capacity (0.2 C) and mass of 5473 cells from the same batch, (**b**) the correlation between cell capacity and mass.

Figure 1(a) shows the distribution of capacity and mass of 5473 cells. The mean values of cell capacity and mass are 5.41 Ah and 92.0 g, respectively. In addition, significant deviations from the normal distribution can be seen. A multimodal distribution is revealed for both capacity and mass. In the literature, deviations from the normal distribution are also found^9^,^10^. Figure 1(b) shows a linear correlation between the cells’ capacity and mass. Since the cells’ capacity was measured with a low rate of 0.2 C at room temperature, the thermodynamic factors dominate the cell capacity while the kinetic influence is minor. As a result, the cells’ capacity is largely determined by the cells’ mass.

### Rates dependence of CtCVs: evidence from 198 cells

We set out to explore how the distribution of capacity, the distribution of direct current resistance (DCR), the capacity-mass correlation and the capacity-DCR correlation vary as a function of the rate. On one hand, as mentioned above, the cell mass is an easily-accessible descriptor corresponding to the thermodynamic factors. On the other hand, the DCR is a widely-used descriptor of kinetic factors. As a result, the rate dependence of the capacity-mass and capacity-DCR correlations can reflect the changes of thermodynamic and kinetic contributions to the CtCVs as a function of the rate.

**Figure 2:**
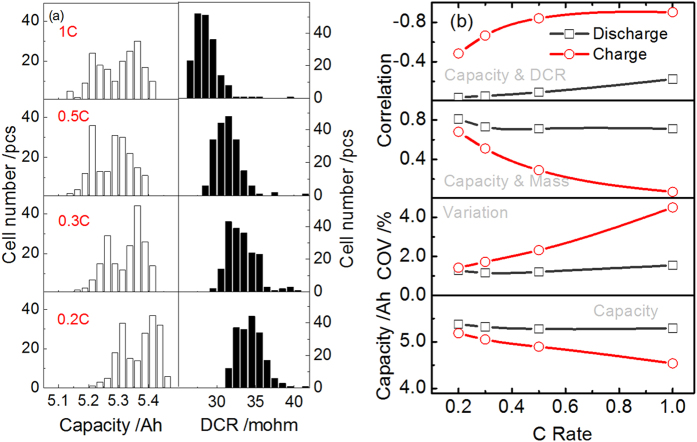
(**a**) Distributions of capacity and DCR (0% SOC) of 198 cells at four rates: 0.2 C, 0.3 C, 0.5 C and 1.0 C during discharge at 25 °C; (**b**) from bottom to top: the mean value of capacity, the CtCVs in terms of capacity, the capacity-mass correlation, and the capacity-DCR correlation, respectively, as a function of the rate during both charge and discharge.

Figure 2(a) shows the capacity and DCR distributions of 198 cells at four discharge rates: 0.2 C, 0.3 C, 0.5 C and 1 C, respectively. Being different from the capacity distribution, the DCR distribution can be approximately described by a normal distribution. Both the capacity and DCR distributions shift to smaller values with increasing the rate. The decreasing of cell capacity with increasing the rate is straightforward. The negative correlation between the DCR and the rate can be inferred from the Butler-Volmer equation, as detailed in ref. 11, 12, 13.

From bottom to top, Fig. 2(b) depicts the mean value of capacity, the CtCVs in terms of capacity, the capacity-mass correlation, and the capacity-DCR correlation, respectively, as a function of the rate during both charge and discharge. The findings are: (1) the CtCVs in terms of capacity become greater with increasing the rate; (2) the magnitude of the coefficient of the capacity-mass correlation decreases with increasing the rate; (3) the magnitude of the coefficient of the capacity-DCR correlation increases with increasing the rate. As a result, we conclude that the capacity as well as the CtCVs are dominated to a greater extent by kinetic factors with increasing the rate. In addition, remarkable differences between charge and discharge can be noticed in Fig. 2(b). This phenomenon can be understood by the virtue of the incremental capacity analysis (ICA) curve, which is provided in supporting information.

A simple phenomenological model was developed to understand the above experimental results. Here we just outline the basic idea of the model, and more details about the development and simulation of the model are found in the supporting information. The cells’ capacity is obtained by integrating the current over the operating time. For a constant current charg/discharge, the capacity is determined by the charge/discharge time. Let us take the discharge as an example. The discharge current is interrupted at a certain time (SOC) when the cell voltage meets the lower voltage limit, viz. 2.5 V. The cell voltage is given by, 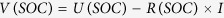, where *U*(*SOC*) is the open-circuit voltage as a function of the SOC which is given by, 
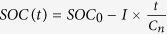
, *R*(*SOC*) is the DCR at the special SOC and *I* is the discharge current. In this model, the thermodynamic factors are built into *U*(*SOC*). Specifically, the nominal capacity *C*_*n*_ is assumed to be proportional to the cell mass *M*_cell_. The kinetic factors are embodied in the term *R*(*SOC*)×I. The cell capacity *C*_I_, is given by, 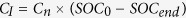 wherein the *SOC*_0_ and *SOC*_*end*_ correspond to the SOC at the beginning and end of the discharge process, respectively. The validity of this model has been scrutinized in ref. 14 by examining the relation between *C*_*I*_ and *I*.

In the simulation, we generated normal distributions of *M*_cell_ and DCR with preset mean values and variations as model inputs, listed in Table S1. Using the Monte Carlo method, we can calculate capacities of these cells with the preset distribution information based on the above simple model. Then, the mean value and CtCVs of capacity among these cells can be obtained. 13,000 cells were simulated and the simulation was repeated for 50 times to calculate the error range in the simulation.

**Figure 3:**
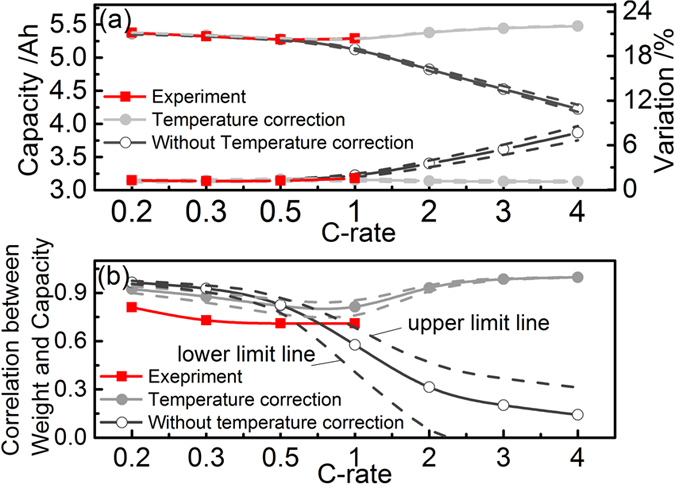
Comparison of experimental and modelling results in terms of (**a**) discharge capacity with different discharge rates; (**b**) the capacity-mass correlation during discharge process at room temperature. The simulation error range is indicated with dotted lines, including the upper and lower limits, and the data indicated with solid line is the average of these 50 simulation results.

Figure 3(a) shows that the cell capacity decreases while the CtCVs increase with increasing the rate. Figure 3(b) exhibits the simulated capacity-mass correlation as a function of the rate. Consistent with the experimental results, the magnitude of the coefficient of the capacity-mass correlation decreases with increasing the rate. We find that when the rate is higher than 1 C, the thermal effects must be considered. A higher temperature rise reduces the kinetic losses, leading to a capacity gain and an increase in the coefficient of the capacity-mass correlation at 1 C compared to 0.5 C. An empirical relation correlating the temperature rise with the rate was used in this model, and more comprehensive models can be integrated for a more accurate simulation^15^.

### Deconvolution of kinetic factors: impedance of eight cells

Given that the kinetic factors play a more and more significant role in the CtCV with increasing the rate, it is crucial to single out the dominating factors of kinetic factors, which is the aim of this section.

**Figure 4:**
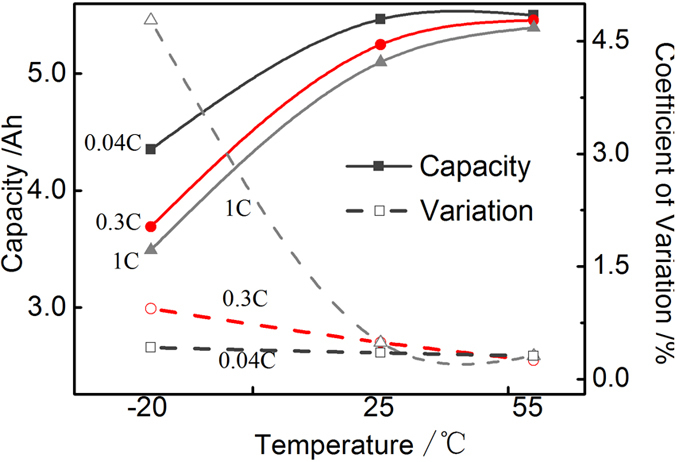
Average discharge capacity and the coefficient of variation at different rates for different temperatures.

It is important to point out that temperature, in addition to the rate, is another vital factor in dictating the relative magnitude of thermodynamic and kinetic factors. Figures 4 and S1 show the discharge characteristics for eight cells at different rates for different temperatures. At 1 C, the average discharge capacity at −20 °C is 68.5% of that at 25 °C, while the value is 80% for 0.04 C. The coefficient of variation (COV) at 0.04 C under 25 °C is very small, 0.35%, and increasing the temperature to 55 °C doesn’t lead to a further reduction in the COV. Hence, one can conclude that kinetic effects, or polarization, can be neglected for such a low rate at temperature above 25 °C. In other words, the CtCVs under 0.04 C at 25 °C are primarily attributed to inherent thermodynamics of these cells. The COV at 1 C under −20 °C is 4.78%, however, it is 0.49% under 25 °C. It is clearly indicated that, at a medium rate such as 1 C, the CtCVs is exaggerated by lowering the temperature, which is ascribed to kinetic factors which are heavily dependent on temperature through an exponential type Arrhenius equation.

The kinetic processes involved in lithium-ion batteries can be broadly divided into three categories: (1) Ohmic process such as electric connection, contact resistance between different layers in the cell; (2) interfacial processes such as lithium-ion transfer across the solid /electrolyte interphase (SEI); (3) bulk diffusion processes such as lithium-ion diffusion in the active particles (solid phase) and the electrolyte (solution phase). For a comprehensive understanding of the physico-chemical processes in LIC electrodes, readers are suggested to refer to theoretical studies such as in refs 4 and 16. This section, assisted by various impedance measurement methods, aims at giving a further decomposition of kinetic contributions in the CtCVs.

Figure S2 shows the difference between DEIS and SEIS. The DEIS and SEIS are almost identical at 25 °C and 55 °C in the frequency range of 10 kHz to 1 Hz. However at −20 °C, the size of semicircles of the DEIS is significantly smaller than that of the SEIS, which is mainly attributed to the remarkable temperature rise during the DEIS measurement concurrently with dynamic charge and discharge. As a result, DEIS method is irreplaceable when one wants to capture the CtCVs under operating conditions at low temperatures. On the contrary, at high temperatures, one can equivalently use SEIS to characterize the CtCVs. Figure S3 displays the DEIS (−20 °C) and SEIS (25 and 55 °C) for eight cells at 10% SOC. To obtain a quantitative analysis, we fitted the impedance spectrum using an equivalent electric circuit model. The fitted resistances, including the ohmic resistance *R*_s_, film resistance *R*_SEI_, charge transfer resistance *R*_ct_, are shown in Figure S4.

**Figure 5:**
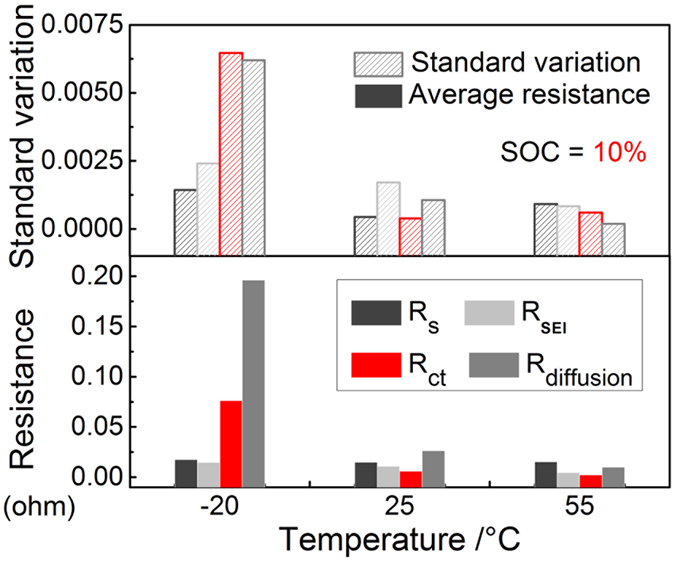
The magnitude of and absolute variation in the various constituent resistances as a function of temperature at 10% SOC.

The total resistance, *R*_*total*_, consists of *R*_*s*_, *R*_*SEI*_, *R*_*ct*_ and the diffusion resistance *R*_*diffusion*_. As demonstrated above, *R*_*s*_, *R*_*SEI*_, *R*_*ct*_ can be obtained through fitting the EIS data using an equivalent electric circuit model. In addition, *R*_*total*_ is calculated from the DCR method during discharge as, 
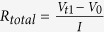
, where *V*_0_ is the voltage at the moment the discharge current *I* is interrupted, *V*_*t*1_ is the voltage after a rest time of *t*_1_ = 1620 s. As a consequence, *R*_*diffusion*_ is calculated by subtracting *R*_*s*_, *R*_*SEI*_ and *R*_*ct*_ from the total resistance *R*_*total*_. The results of these four types of resistance are shown in Figs 5 and S4. *R*_*s*_ and *R*_*SEI*_ change little with temperature for all the evaluated SOCs. *R*_*ct*_ and *R*_*diffusion*_ increase greatly as the temperature decreases. Specifically, *R*_*ct*_ increases seven times at −20 °C compared to the value at 25 °C, while *R*_*diffusion*_ increases 15 times in the same temperature change. At −20 °C, *R*_*diffusion*_ occupies nearly 64.5% of *R*_*total*_, while *R*_*ct*_ makes a contribution of 25%. Note that they keep nearly the same at high temperatures such as 25 °C and 55 °C. The absolute variation in *R*_*ct*_ and *R*_*diffusion*_ are larger than other two resistances, *R*_*s*_ and *R*_*SEI*_, especially at lower temperatures. It is then concluded that, among the four kinds of kinetic processes, the charge transfer at the electrode/electrolyte interfaces and the diffusion of ions in active particles and the electrolyte account for the majority of CtCVs originating from kinetic factors.

## Discussion

We have demonstrated that the rate has a strong effect on CtCVs, and we try to understand this rate dependence of the CtCV from thermodynamic and kinetic factors. The contribution of thermodynamic factors, reflected by the magnitude of the coefficient of the capacity-mass correlation, is weakened with increasing the rate. In contrast, the contribution of kinetic factors, embodied by the magnitude of the coefficient of the capacity-DCR correlation, is intensified with increasing the rate.

In practice, the batteries in battery electric vehicles (BEVs) work at a relatively small current, which is usually less than 0.2 C. However, the batteries in plug-in hybrid electric vehicles (PHEVs) and hybrid electric vehicles (HEVs) work at a much higher rate, for example, 5 C. In this regard, our conclusion implies that we should take different strategies to improve the CtCVs for LICs in different types of EVs. Table 1 lists the critical parameters which contribute to the thermodynamic and kinetic parts of LICs. Table 2 lists the controlled parameters of the LICs in the production processes. Based on the above analysis, for batteries used in BEVs, more attention should be paid to the constituent and loading density of active materials to control the CtCVs. In contrast, regarding batteries used in PHEVs and HEVs, it would be more effective to improve the CtCVs by putting more stringent control on the porosity, tortuosity, conductivities, and diffusivities of the electrode^17^,^18^,^19^,^20^,^21^.

## Conclusions

In summary, this study dwells upon the cell-to-cell variations of lithium-ion batteries. Statistical analysis of 5473 cells prior to the cell-screening process from a same batch reveals that the cell capacity measured at a small rate of 0.2 C is nearly linear with the cell weight. Increasing the rate from 0.2 C to 1.0 C decreases the magnitude of the coefficient of capacity-mass correlation, while leading to a greater magnitude of the coefficient of capacity-DCR correlation. We conclude that the kinetic factors, of which the DCR is a common descriptor, play a more and more important role with increasing the rate. Combined steady and dynamic impedance analysis show that the charge transfer process at the electrode/electrolyte interfaces and the diffusion of ions in active particles account for the majority of kinetic factors. A simple model has been developed for the analysis of the rate dependence of cell-to-cell variations.

## Methods

Swing 5300 cells from a battery manufacturer Boston-power (BPI) with a nominal capacity of 5.3 Ah (discharge with 0.2 C at 23 ± 2 °C) were used. The positive electrode is the blend of LiNi_0.8_Co_0.15_Al_0.05_O_2_ (NCA) and LiNi_0.5_Co_0.3_Mn_0.2_O_2_ (NCM). The negative electrode is the artificial graphite. The separator is polypropylene (PP). The electrolyte is the mixture of ethylene carbonate (EC), ethyl methyl carbonate (EMC), and dimethyl carbonate (DMC), containing lithium hexafluorophosphate (LiPF_6_).

Firstly, we collected the data of capacity (discharge at 0.2 C and at 23 ± 2 °C) and mass of 5473 cells from the same batch. Secondly, we singled out 198 cells and explored the rate capability by testing their capacities at 0.2 C (1.06 A)/0.3 C (1.59 A)/0.5 C (2.65 A)/1 C (5.3 A) during both charge and discharge at 23 ± 2 °C. In terms of rate capability during charge, the constant current (CC) charge protocol with a voltage limit of 4.2 V was used, followed by the discharge at 0.3 C to 2.5 V. In terms of rate capability during discharge, the cells were charged at 0.3 C with the CC-CV (constant current - constant voltage) protocol to 4.2 V and a cutting-off current of 0.159 A, and then the cells were discharged to 2.5 V with different rates. In addition, the direct current resistance (DCR) was calculated through dividing the over-potential at the end of discharge/charge with respect to the current. The electrochemical tests on the eight cells are detailed below.

### Capacity test protocol

The capacities were measured at three different temperatures, −20 °C, 25 °C and 55 °C, with three different discharge rates, 0.04 C (0.21 A), 0.3 C (1.59 A), and ~1 C (5 A), respectively. The experiments were conducted using the Maccor MC-8 (5 V/5 A) with ambient temperature controlled by a high-low temperature chamber. Meanwhile, the temperature on the cell surface was monitored with thermocouples, and the data were recorded by using the Agilent-34970.

We first calibrated the capacities of these cells with 0.3 C for both charge and discharge at 25 °C: The charge protocol was CC-CV with an upper voltage limit of 4.2 V, and the cutting-off current was 0.02 A. After a rest period of 10 min, the cells were discharged with a constant current (0.3 C) to 2.5 V. This charge-discharge cycle was repeated 3 times. Then, we started below test protocols: (1) the cells were charged to 100% state of charge (SOC) with 0.3 C at 25 °C using the same charge protocol with the calibration process; (2) the cells were adjusted to a certain temperature among 25 °C, 55 °C and −20 °C. It was noted that the cells were stabilized at each temperature for 4 hrs in the high-low temperature chamber; (3) the cells were discharged at a certain rate to 2.5 V under the temperature preset in step 2; (4) the chamber temperature was adjusted to 25 °C. The cells were recovered by charging and discharging for 3 times using the same charge protocol as in the calibration process; (5) steps 1 to 4 were repeated for each discharge rate: 0.04 C, 0.3 C, 1 C at the preset temperature; (6) steps 1 to 5 were repeated at 25 °C, 55 °C and −20 °C.

### Impedance test methods

In terms of EIS, we used static EIS (SEIS, that is, the impedance was measured when the cells were at steady state) to analyze the impedance at temperatures higher than 25 °C, and dynamic EIS (DEIS, that is, the impedance was measured when the cells were during charge/discharge with a finite direct current (DC)) to analyze the impedance at lower temperatures such as −20 °C^11^,^22^. To examine the CtCVs under operating conditions, we selected the DEIS method which was capable of collecting the EIS during charge/discharge. We conducted the SEIS at temperatures over 25 °C instead, because the differences between SEIS and DEIS are sufficiently small at these temperatures and the SEIS has the merits of a broader frequency range and superior stability at low frequencies. The frequency range for SEIS was 10 kHz to 0.01 Hz, and the amplitude of the stimulating current was 0.1 A. However, the frequency range for DEIS was 10 kHz to 0.1 Hz. It was noticed that the lower frequency limit of the DEIS was one-decade higher than that of the SEIS because stimulus at lower frequencies would induce more significant SOC changes due to the DC in the DEIS, thus leading to the violation of the stability criterion of impedance measurement^22^. In a companion study, we had a detailed discussion on this topic^22^. The discharge current in the DEIS test was 2 A and the amplitude of the stimulating current was 0.5 A.

## Additional Information

**How to cite this article**: An, F. *et al*. Rate dependence of cell-to-cell variations of lithium-ion cells. *Sci. Rep*. **6**, 35051; doi: 10.1038/srep35051 (2016).

## Supplementary Material

Supplementary Information

## Figures and Tables

**Table 1:** Critical parameters affecting the thermodynamic and kinetic.

Attribute	Critical parameters
Thermodynamic	The mass fraction of active material
	Loading density
Kinetic	Electrode porosity and tortuosity
	Electrolyte conductivity
	Charge transfer kinetics
	Diffusion coefficient of lithium ions in the electrolyte phase and active materials

**Table 2:** The correspondence between controlled parameters and production processes.

Process	Controlled parameters
Mixing	The constitute of components
	The mass fraction of active material
Coating	Loading density
Calendaring	Electrode thickness
Slitting	Electrode adhesion
	Electrode width
Assembling	Electrolyte quantity
	Electrolyte conductivity
Formation – Charge/discharge	Environment temperature
	Charge – discharge current
