# Supplementary material for: Rate dependence of cell-to-cell variations of lithium-ion cells
Source: Sci Rep. 2016 Oct 11;6:35051. doi: 10.1038/srep35051 (PMC5057166; doi:10.1038/srep35051)
Supplement: Supplementary Information [file srep35051-s1.doc]

Supporting information

Rate dependence of cell to cell variations of lithium-ion cells

Fuqiang An^1,2^, Lufan Chen^2^, Jun Huang^3^, Jianbo Zhang^3,4,*^, Ping Li^1,**^

1. Institute for Advanced Materials and Technology, University of Science and Technology Beijing, Beijing 100083, China;

2. Boston-Power Battery, Inc. Westborough, USA

3. State Key Laboratory of Automotive Safety and Energy, Department of Automotive Engineering, Tsinghua University, Beijing 100084, China

4. Beijing Co-innovation Center for Electric Vehicles, Beijing Institute of Technology, Beijing 100081, China

Corresponding authors: jbzhang@mail.tsinghua.edu.cn (J. Zhang), liping@ustb.edu.cn (P. Li)

|  |
| --- |
| (a) |
| Figure S1. Discharge characteristics at different rates for different temperatures |

|  |
| --- |
| (a) |
|  |
| (b) |
|  |
| (c) |
| Figure S2. Comparison between SEIS and DEIS at 10% SOC at three temperatures: (a) 25 °C, (b) 55 °C and (c) -20 °C. "D” and “C” represent discharge and charge, respectively. |

|  |
| --- |
| (a) |
|  |
| (b) |
|  |
| (c) |
|  |
|  |
| (d) |
| Figure S3. The DEIS and SEIS for 8 cells at 10% SOC at different temperatures: (a) 25°C, (b) 55°C, (c) -20°C. (d) The equivalent electric circuit model for data fitting. |

|  |
| --- |
| (a) |
|  |
| (b) |
| Figure S4. The magnitude of and absolute variation in the various constituent resistances as a function of temperature at different SOCs: (a) 50%, (b) 90%. |

**Distinction between charge and discharge process**

Fig.2(b) shows that the characteristics during charge process is more sensitive to the C rate than those during the discharge process. A key underlying cause is the different capacity changing rates with respect to the voltage at the end of charge and discharge. Fig.S5 shows the ICA curve of cell and it will be used in our explanation below.

Figure S5. Incremental capacity analysis (ICA) curve

The capacity variation can be easily calculated in Fig.S5 by the integral area between dQ/dV curve and baseline (the grey line), which can be expressed as below:

$$\Delta C=\int_{V_{cut-off}-I_{1}R_{1}}^{V_{cut-off}-I_{2}R_{2}} \frac{dQ}{dV}dV$$

Wherein I_1_R_1_ and I_2_R_2_ represent two different discharge (I:-) / charge (I:+) situation, which include but are not limited to: 1) the same cell discharge / charge under different currents (R_1_=R­_2_, corresponding to the capacity changing rate as different currents); 2) different cell discharge / charge under the same current (I_1_= I_2_, corresponding to the variation of capacity under different currents).

Refer to the curve in Fig.S5, we can easily find the capacity changing rate with voltage (dQ/dV) is smaller at the end of discharge (2.75V) than that of charge (4.2V), which means the capacity difference of charge process is more sensitive to the voltage difference (I_2_R_2_ - I_1_R_1_) than that of discharge process, which is consistent with the experimental results shown in Fig.2(b).

**The development of the model used in simulation**

- 1. Model development

At the end of constant discharge of Li-ion cells, the correlation between voltage and *OCV* can be expressed as below:

$V_{\min}=OCV-R_{cell}I$ (1)

Wherein, *V_min_*- cutoff voltage during discharge (2.5V in this study), *OCV* – open circuit voltage, *R_cell_* – over potential resistance, and I – discharge current.

According to the *SOC-OCV* curve, there is an approximately linear relationship between *SOC* and *OCV* at low *SOC*, which can be expressed as follow:

$OCV=c_{1}SOC+c_{2}$ (2)

The constant *c_1_* and *c_2_* here are the fitting coefficients.

Combined with the *SOC* definition$SOC=1-\frac{C_{d}}{C_{n}}$ , wherein *C_d_* is the discharging capacity and *C_n_* is the nominal capacity, the relation between discharging capacity and over-potential resistance can be deduced:

$C_{d}=\frac{C_{n}}{c_{1}}(c_{1}+c_{2}-V_{\min})-\frac{C_{n}}{c_{1}}R_{cell}I$ (3)

In previous discussion, we can observe a linear relationship between nominal capacity *C_n_* and cell Mass *M_cell_*, and the equation above can be expressed as:

$C_{d}=\frac{c_{3}M_{cell}+c_{4}}{c_{1}}(c_{1}+c_{2}-V_{\min})-\frac{c_{3}M_{cell}+c_{4}}{c_{1}}R_{cell}I$ (4)

Similar with *c_1_* and *c_2_*, the constant *c_3_* and *c_4_* here are the fitting coefficients.

In equation (4), *C_n_* is the normal capacity, which is decided by the active material quantity of single cell, whereas *R_cell_* is the over-potential resistance varied as the temperature changed and cell variation. According to our former results, the temperature rise caused by discharge cannot be ignored when the current *I* exceeded *I_0_*, the critical current, decided by the surrounding temperature and the thermal properties of cells. In this study, we have to consider and correct the change of resistance caused by temperature rise since the current reached 1C or higher.

The resistance *R_cell_* is inversely proportional to reaction rate *k*, that is:

$\begin{matrix} Arrhenius\text{ }equation:k=Ae^{-\frac{E_{A}}{RT}} \\ \end{matrix}$ (5)

$R_{cell}\propto\frac{1}{k}\propto e^{\frac{E_{A}}{RT}}$ (6)

Wherein, *E_A_* is the activation energy, *T* is temperature and *R* is constant. According to our previous research, there is an approximate linear relationship between discharging current *I* and the cell temperature at the end of discharge, which is corresponding to *R_cell_*, the over-potential resistance at the end of discharge, that is:

$T=mI+n$ (7)

$R_{cell}=e^{\frac{E_{A}}{(mI+n)R}-\frac{E_{A}}{nR}}\cdot R_{cell,0}=Kt(I)\cdot R_{cell,0}$ (8)

Here, the constant *m* and *n* are the fitting coefficients, *R_cell,0_* is the over-potential resistance without temperature rise, and *Kt(I)* is the coefficient for temperature correction. Therefore, equation (4) can be expressed as below:

$C_{d}=\frac{c_{3}M_{cell}+c_{4}}{c_{1}}(c_{1}+c_{2}-V_{\min})-\frac{c_{3}M_{cell}+c_{4}}{c_{1}}R_{cell,0}Kt(I)I$ (9)

- 1. Determination of model parameters

From the formula above, we can calculate the discharging capacity of different cells under different currents as long as the parameters (such as c_1_, c_2_, etc.) are provided. Fortunately, the value of most parameters in formula (9) can be obtained through experiment, measurement or curve fitting. The method for obtaining parameter value is described hereinafter, and value of the parameters used in this paper has been listed in Table.S1 for the readers’ reference.

As mentioned above, c_1_ and c_2_, c_3_ and c_4_, as well as m and n are the linear fitting coefficients of SOC-OCV curve, cell mass – capacity relationship, and current – temperature curve respectively, which can be obtained through curve fitting based on the experimental results.

Since the cells are sampled from the production line directly, we can obtain the mass of each cell and thereby the average and variation of them. In the manuscript, we have presented the capacity and variation of 198 cells under four different currents. Given these experimental data, activation energy E_A_ and the resistance *R_cell,0_* of every cell as well as their variation can be fitted combined with other parameters.

- 1. Monte Carlo Simulation

In the equation (9), only *M_cell_* and *R_cell_* vary from cell to cell and follow normal distribution, which is:

$$M_{cell,i}\sim N(M_{cell},\delta_{M}^{2}),R_{cell,i}\sim N(R_{cell},\delta_{R}^{2})$$

The activation energy E_A_ also varies from cell to cell, and the average value of 198 cells is used here for simplified calculation.

Given parameter obtaining method mentioned earlier, the discharging capacity of over 13,000 cells are generated in computer with the mass and resistance respectively following the normal distribution above, and their variation of capacity under different discharging current are calculated and shown.

Moreover, the correlation *r* between cell mass *M_cell_* and discharging capacity *C_d_* can be calculated through the formula below, which will vary as discharging current *I*:

- 1. Simulation results

Fig.3(a) shows the curve fitting results, including the average and variation of capacity, which also confirmed the necessity of temperature correction, and Fig.3(b) shows the correlation *r* between cell mass *M_cell_* and discharging capacity *C_d_* under different currents.

The simulation was repeated for 50 times to calculated the simulation error range, which is indicated with dotted line, including the upper and lower limit, and the data indicated with solid line is the average of these 50 times simulation results.

The experimental results of 198 cells is also showed in Fig.3 for comparison, indicated with red line and mark.

Table S1 Parameters used in the model simulation

| V_min_ | 2.75 V | cutoff voltage during discharge |
| --- | --- | --- |
| c_1_ | 9.50 | fitted coefficients of SOC-OCV curve |
| c_2_ | 2.98 | fitted coefficients of SOC-OCV curve |
| R | 8.314 J/（mol·K） | gas constant |
| E_A_ | 58.55 kJ/mol | fitted activation energy |
| c_3_ | 0.137 | fitted coefficients of M_cell_-Capacity relation |
| c_4_ | -7.259 | fitted coefficients of M_cell_-Capacity relation |
| m | 3.961 | fitted coefficients of Current Rate-Temperature relation |
| n | 295.77 | fitted coefficients of Current Rate-Temperature relation |
| M_cell_ | 92.11 g | cell mass |
| δ_M_ | 1.01 | standard variation of M_cell_ |
| R_cell,0_ | 0.274ohm | cell resistance, fitted but close to the experimental results |
| δ_R_ | 0.05 | standard variation of R_cell,0_ |
